# Impaired chondrocyte U3 snoRNA expression in osteoarthritis impacts the chondrocyte protein translation apparatus

**DOI:** 10.1038/s41598-020-70453-9

**Published:** 2020-08-10

**Authors:** Ellen G. J. Ripmeester, Marjolein M. J. Caron, G. G. H. van den Akker, Don A. M. Surtel, Andy Cremers, Panagiotis Balaskas, Philip Dyer, Bas A. C. Housmans, Alzbeta Chabronova, Aibek Smagul, Yongxiang Fang, Lodewijk W. van Rhijn, Mandy J. Peffers, Tim J. M. Welting

**Affiliations:** 1 Laboratory for Experimental Orthopedics, Department of Orthopedic Surgery, Maastricht University, Universiteitssingel 50, 6229 ER Maastricht, The Netherlands; 2 Laboratory for Experimental Orthopedics, Department of Orthopedic Surgery, Maastricht University Medical Center, P.O. Box 5800, 6202 AZ Maastricht, The Netherlands; 3 Department of Musculoskeletal Biology, Institute of Ageing and Chronic Disease, University of Liverpool, Liverpool, L69 3BX UK

**Keywords:** Mechanisms of disease, Small RNAs

## Abstract

Although pathways controlling ribosome activity have been described to regulate chondrocyte homeostasis in osteoarthritis, ribosome biogenesis in osteoarthritis is unexplored. We hypothesized that U3 snoRNA, a non-coding RNA involved in ribosomal RNA maturation, is critical for chondrocyte protein translation capacity in osteoarthritis. U3 snoRNA was one of a number of snoRNAs with decreased expression in osteoarthritic cartilage and osteoarthritic chondrocytes. OA synovial fluid impacted U3 snoRNA expression by affecting U3 snoRNA gene promoter activity, while BMP7 was able to increase its expression. Altering U3 snoRNA expression resulted in changes in chondrocyte phenotype. Interference with U3 snoRNA expression led to reduction of rRNA levels and translational capacity, whilst induced expression of U3 snoRNA was accompanied by increased 18S and 28S rRNA levels and elevated protein translation. Whole proteome analysis revealed a global impact of reduced U3 snoRNA expression on protein translational processes and inflammatory pathways. For the first time we demonstrate implications of a snoRNA in osteoarthritis chondrocyte biology and investigated its role in the chondrocyte differentiation status, rRNA levels and protein translational capacity.

## Introduction

Osteoarthritis (OA) is a chronic debilitating joint disease that is strongly associated with ageing^1,2^. OA involves pathological cellular processes in all joint structures and affects articular cartilage integrity, leading to dysfunctional joint articulation^2^. During OA development and progression, the articular chondrocyte’s phenotype changes^3–5^ and presents with disturbed cellular homeostasis characterized by abnormal expression of (pre-)hypertrophic-[*RUNX2 *(*runt-related transcription factor 2*); *COL10A1 *(*type X collagen*)], catabolic-[*ALPL *(*alkaline phosphatase*);* MMP13* (*matrix metallopeptidase 13*) and *ADAMTS5 *(*a disintegrin and metalloproteinase with thrombospondin motifs 5*) and inflammatory (*COX2* (*cyclooxygenase 2*) and *IL-6 *(*interleukin 6*)] genes, while chondrogenic gene expression [*SOX9* (*SRY-box transcription factor 9*);* COL2A1* (*type 2 collagen*); *ACAN* (*aggrecan*) and *NKX3-2* (*NK3 homeobox 2*)] is attenuated^3,4^. The biomolecular processes that catalyze disturbances in the articular chondrocyte phenotype leading to OA are poorly understood, and it is expected that a comprehensive understanding of the avenues leading to disruption of articular chondrocyte homeostasis will provide important clues for future treatments.

Chondrocytes are specialized secretory cells, enabling the synthesis and maintenance of the protein-rich cartilage extracellular matrix (ECM). Disturbances in chondrocyte protein translation in cartilage development and OA are connected to mTOR (mammalian target of rapamycin) activity^6^, endoplasmic reticulum stress^7^, unfolded protein response and apoptosis^8^. These responses change the downstream translational activity of the biosynthesized ribosome. The assembled mammalian ribosome is built from ribosomal RNAs (rRNAs), together with at least 80 different protein subunits^9^. In the ribosome the 18S rRNA guides the decoding of the mRNA message, while the 28S rRNA forms the core of the peptidyltransferase center that polymerizes the amino acid sequence encoded by the mRNA into functional proteins. Post-transcriptional maturation of rRNAs is an integral part of the biosynthesis of ribosomes^10–13^ and ribonucleolytic processing of the major 47S rRNA precursor into mature 18S, 5.8S, and 28S rRNAs is rate-limiting for ribosome biogenesis^10^. The U3 small nucleolar RNA (snoRNA) is a highly abundant and evolutionarily conserved box C/D-class snoRNA, guiding the endoribonucleolytic processing of the 5′ external transcribed spacer (ETS) of the 47S pre-rRNA by base complementarity-guided pre-rRNA substrate recognition^14^ and plays a key role in the maturation of 18S rRNA^12,15^. Although extensively studied in yeast^16^, it was only recently demonstrated that U3 snoRNA is indispensable for rRNA maturation in human cells^16^.

Pathways controlling ribosome activity have been described in the regulation of chondrocyte homeostasis^17,18^. Here, we postulated that not only ribosome activity is involved in chondrocyte homeostasis, but that OA pathophysiological situations can cause alterations in chondrocyte ribosome biogenesis with consequences for cellular protein translation. In this study we discovered that the chondrocyte expression of ribosome biogenesis factor U3 snoRNA is impacted in OA cartilage and we investigated its connection with OA pathophysiological conditions in chondrocytes and its role in influencing rRNA levels and chondrocyte translation capacity.

## Materials and methods

### Cell culture

Human articular chondrocytes (HACs) were isolated from femoral cartilage from total knee arthroplasty for end‐stage OA (OA-HACs; average age 68.5 years with SD 8.2 years). Non-OA chondrocytes were isolated from cartilage of non-OA individuals undergoing anterior cruciate ligament repair (average age 25.7 years with SD 17.9 years). Medical ethical approval for collecting and using HACs was received from the Medical Ethics Committee from the Maastricht University Medical Center (approval number 2017-0183). Dutch medical ethical guidelines were followed for studying human samples and informed consent was acquired. Chondrocyte isolation and culture was performed as described previously^5^. SW1353 cells were purchased from ATCC (ATCC; Middlesex, UK) and cultured in a humidified atmosphere at 37 °C, 5% CO_2_. Culture medium was Dulbecco’s Modified Eagle Medium (DMEM)/F-12 (Life Technologies, Waltham, Massachusetts, USA) supplemented with 10% fetal calf serum (FCS; Sigma-Aldrich, Dorset, UK), 1% non-essential amino acids (NEAA; Life Technologies) and 1% penicillin/streptomycin (P/S, Invitrogen Life Technologies). In experiments non-OA chondrocytes were exposed to control (20% (v/v) of 0.9% NaCl), non-OA synovial fluid (non-OA SF; 20% (v/v); pool of ten donors; average age 56.7 years with SD 7.5 years; purchased from Articular Engineering, Northbrook, Illinois, USA) or OA synovial fluid (OA-SF; 20% (v/v); pool of six or ten donors; for ten-donors-pool average age 65.4 years with SD 9.1 years; for six-donor-pool average age 73.0 years with SD 6.8 years; MEC approval 2017-0183). The average age difference between the ten-donor-pools was not statistically significant (Mann Whitney, p = 0.069). SW1353 and non-OA HACs were exposed to BMP7 (R&D Systems, Minneapolis, Minnesota, USA).

### Microarray

Cartilage biopsies were obtained from the femoral intercondylar notch of non-OA male human knees of young individuals undergoing anterior cruciate ligament repair surgery (n = 6; mean age ± SD 22.7 ± 4.1 years). OA cartilage was obtained from male OA patients undergoing total knee arthroplasty (n = 6; mean age 66.4 ± 15.9 years). Sample characteristics; sample collection; RNA isolation of OA and healthy cartilage; the micro-array and data analysis have been performed as previously reported^19^. The heat map of hierarchical clusters of correlations and PCA plot are shown in Supplementary Fig. 1. Medical ethical approval for collecting and using cartilage biopsies was received from the Medical Ethics Committee from the Maastricht University Medical Center (approval number 08-04-028 and 14-4-038). Dutch medical ethical guidelines were followed for studying human samples and informed consent was acquired. Sequence data have been submitted to National Centre for Biotechnology Information Gene Expression Omnibus (NCBI GEO); E-MTAB-5715.

### DMM

Mice (C57BL/6) were group housed in individually ventilated cages at a 12 h light/dark cycle, with ad libitum access to food and water. Under anesthesia, a 3 mm skin incision was made over the medial aspect of the patellar ligament through the joint capsule into the femorotibial joint of the left knee. The medial meniscotibial ligament was transected to destabilize the cranial pole of the medial meniscus from the anterior tibial plateau. In sham-operated mice (n = 3) the medial meniscotibial ligament was visualized but not transected. Mice were sacrificed 8 weeks post-surgery and stifles were fixated in phosphate-buffered 3.7% formalin. Ethical review was conducted by the University of Liverpool Animal Welfare and Ethical Review Body and ethical approval was obtained from the University of Liverpool (project license P74DC0667). All experimental protocols were performed in compliance with the UK Animals (Scientific Procedures) Act 1986 regulations.

### U3 snoRNA in situ hybridization

Dissected stifles were decalcified in a 1:1 10% formalin (VWR, Radnor, Pennsylvania, USA): 200 mM EDTA (VWR) for 3 weeks, embedded in paraffin and sectioned at 5 µm, three adjacent sections per slide. Following dewaxing sections were circled with a hydrophobic pen (Agilent, Santa Clara, California, US). U3 snoRNA in situ hybridization was performed following the manufacturer’s instructions of the miRCURY LNA miRNA ISH Optimization kit (Qiagen, Hilden, Germany). Probe sequences (Eurogentec, Liège, Belgium) are shown in Supplementary Table 1. Detection was performed by ~ 1 h incubation at 37 °C with NBT/BCIP (Roche, Basel, Switzerland). Slides were washed with PBS-Tween (PBS-T; phosphate buffered saline with 0.1% Tween-20), counterstained 5 min 0.05% FastGreen, 5 min 0.05% Safranin O, dehydrated to xylene as standard and mounted with Pertex Mounting Medium.

### Knockdown and ectopic expression of U3 snoRNA

Transfection of non-OA HACs or SW1353 cells (seeded at 30,000 or 20,000 cells/cm^2^, respectively) with 100 nM of U3 snoRNA antisense oligonucleotide (ASO) or a scrambled version thereof (SCR) was performed using HiPerfect (Qiagen) according to the manufacturers' protocol. Non-OA HACs were transfected after 8 h of prior serum starvation, while SW1353 cells were transfected without prior serum starvation. Sequences of U3 snoRNA ASO and SCR (Eurogentec, Liège, Belgium) are shown in Supplementary Table 2. Transfection of non-OA HACs with a U3 snoRNA mini-gene was performed using Fugene6, following the manufacturers’ protocol (Promega, Madison, Wisconsin, USA). The U3 snoRNA mini-gene was synthesized (Genecust, Boynes, France) and cloned into the pUC57 vector. The mini-gene consisted of the endogenous 500 nucleotide sequence upstream of the U3 snoRNA transcription start site, the pre-U3 snoRNA sequence and followed by 250 nucleotides downstream of the transcription termination sequence. The U3 mini-gene construct was transfected at 10 ng plasmid/cm^2^ and because of the low amount of U3 snoRNA mini-gene supplemented with pGluc-basic-2-CMV (NEB, Ipswich, Massachusetts, USA) as carrier plasmid. In control conditions, only carrier pGluc-basic-2-CMV was transfected at equal total plasmid content as compared to the U3 mini-gene transfection.

### U3 snoRNA promoter-reporter assay

The human U3 snoRNA gene promoter sequence was synthesized (Genecust, Boynes, France) from the 500 nucleotide sequence upstream of the U3 snoRNA transcription start site of SNORD3A gene (Ensembl) and cloned into the pNL1.2[NlucP] vector (Promega). pNL1.2[NlucP]_Hs_U3 was transfected into non-OA HACs or SW1353 cells (seeded at 15,000 cells/cm^2^) with Fugene6 and re-seeded 2 h after transfection. U3 snoRNA promoter-reporter cells were exposed to OA synovial fluid (OA-SF; 20% (v/v), pool of six donors), corresponding control (20% (v/v) of 0.9% NaCl), BMP7 (R&D Systems, Minneapolis, Minnesota, USA), IL1β (Life Technologies, Waltham, Massachusetts, USA) or TNFα (Sigma-Aldrich, Dorset, UK). For bioluminescence analysis cells were lysed with lysis buffer (Promega) and promoter-activity was measured with the Nano-Glo Luciferase Assay System (Promega) on a Tristar LB 942 (Berthold, Bad Wildbad, Germany). DNA-content was measured using a SYBR-GREEN (Invitrogen) assay. Relative differences were determined as compared to control conditions following correction for background and normalization by DNA content.

### Gene expression

Cells were rinsed with 0.9% NaCl and cell lysis, RNA isolation and subsequent cDNA synthesis were performed using the Cells-to-Ct kit (ThermoFisher Scientific, Waltham, Massachusetts, USA). Alternatively, cells were disrupted with TRIzol reagent (Life Technologies), followed by RNA isolation and quantification and cDNA synthesis as described previously^20^. The real-time quantitative polymerase chain reaction was performed as described previously^5^ and using validated primer sequences described in Supplementary Table 3. Ct-values were analyzed with the standard curve method and RNA (including non-coding- and messenger-RNA) expression was normalized to cyclophilin expression or the relative DNA-content between treatments. DNA-content for normalization was measured in parallel wells receiving the identical treatment. DNA-content was measured using a DAPI and HOECHST staining further explained in “SUNsET-assay”. Differential gene expression was determined as a fold change compared to control conditions.

### In-solution tryptic digestion and mass spectrometry proteomics

Cell lysates following U3 snoRNA knockdown (or control) in SW1353 cells were used^21^. Cells were harvested from triplicate wells of a six well plate with parallel wells used for confirmation of U3 snoRNA knockdown. In-solution tryptic digestion on 10 μg protein was undertaken as previously described^22^. For the secretomes in-solution tryptic digestion was carried out on 10 µl of StrataClean resins (Agilent Genomics, 400,714) on 100 µg of protein for each sample as described previously^23^. Liquid chromatography-tandem mass spectrometry (LC–MS/MS) analysis was performed on trypsin digests using an Ultimate 3,000 Nanosystem (Dionex, ThermoFisher Scientific) online to a Q-Exactive Quadrupole-Orbitrap instrument (Thermo Scientific)^24^. Proteins were identified using an in‐house Mascot server (Matrix Science, London, UK). Search parameters used were as follows: enzyme; trypsin, peptide mass tolerances 10 ppm, fragment mass tolerance of 0.01 Da, 1+, 2+, and 3+ ions, with carbamidomethyl cysteine as a fixed modification and methionine oxidation as a variable modification, searching against the UniHuman Reviewed database, with a false discovery rate (FDR) of 1%, a minimum of two unique peptides per protein.

### Label-free quantification of mass spectrometry proteomics data

Progenesis QI software (version 4, Waters, Manchester, UK) was used to identify fold changes in protein abundance between U3 snoRNA knockdown and scrambled ASO control condition^25^. Only unique peptides were used for quantification, and with p-values < 0.05, were considered to be differentially expressed (DE). Proteomic data has been deposited in the PRIDE ProteomeXchange and can be accessed using the identifier PXD017253^26^.

### Ingenuity pathway analysis

Functional analysis of differentially expressed proteins following U3 knockdown in SW1353 cells was undertaken to evaluate the differences in protein abundance. Ingenuity pathway analysis has been performed as previously described^27^.

### Protein translation capacity

For a puromycilation assay^28,29^, cultures were incubated for 15 min with 5.4 µM puromycin (Sigma-Aldrich, Dorset, UK), immediately followed by washing steps with PBS and fixation for 20 min with 10% formalin (VWR). Wells were washed with PBS-T and treated for 10 min with 0.1% Triton X-100. Wells were blocked for 1.5 h with 1% (m/v) skimmed milk powder (ELK, Campina, Zaltbommel, the Netherlands) in PBS-T, followed by overnight incubation at 4 °C with the anti-puromycin antibody 12D10 (Sigma-Aldrich, Dorset, UK). After washing with PBS-T, wells were incubated for 1 h at room temperature with goat anti-mouse Alexa488 (ThermoFisher Scientific). Following a final wash step, the fluorescence signal intensity was determined using a Tristar LB942 (Berthold, Bad Wildbad, Germany) equipped with excitation filter F485 and emission filter F353. Fluorescence data were normalized to DNA-content^30^. For that, the same wells were subsequently washed with HEPES-Buffered Saline (HBS), followed by 1 h incubation with 5 µg/mL DAPI (Invitrogen) plus 5 µg/mL HOECHST 33342 (Invitrogen) in HBS. After washing steps with HBS, fluorescence signal intensity was determined using a Tristar LB942 (Berthold), using the excitation filter F355 and emission filter F460.

### RNA electrophoresis and northern blotting

Two micrograms total RNA was separated on a 6% PAA-gel (6% PAA (Acrylamide/Bis-acrylamide 30:1); 1 × TBE (Tris–borate-EDTA; Merck, Darmstadt, Germany); 8 M urea), electro-transferred to a Hybond-N membrane (GE Healthcare; Chicago, Illinois, USA) and immobilized by UV-crosslinking (CL-1000, UVP). Membrane was pre-hybridized for 1 h at 62 °C under agitation in hybridization buffer (2 × SSC, 5 × Denhardts solution, 200 μg/ml fish sperm DNA, 0.1% SDS). Membrane was subsequently hybridized overnight with the U3 snoRNA probe (5 nM; Supplementary Table 4) at 62 °C under agitation. Non-hybridized probe was rinsed from the membrane using a series of SSC-SDS wash buffers. Membrane was then incubated in blocking buffer [1 × PBS, 0.5% SDS, 0.1% I-Block (ThermoFisher Scientific)] for 30 min, followed by 30 min incubation in blocking buffer containing 0.2 μg/ml Streptavidin-AP (Life Technologies). After washing with PBS the membrane was equilibrated with detection buffer (100 mM Tris–HCl pH 9.5; 100 mM NaCl; 1 mM MgCl_2_) and detection was carried out using CDP-Star (Sigma-Aldrich, Dorset, UK) and visualized/quantified using a Bio-Rad Chemidoc MP imaging system. U6 snRNA was detected as a reference RNA on the same membrane by reprobing and repeating the procedure in-full while using the U6 snRNA probe. Full length images of northern blots are shown in Supplementary Fig. 4. The U3 snoRNA signal was normalized by the signal for U6 snRNA and relative differences between conditions were calculated.

### Statistical analyses

Statistical significance has been calculated using GraphPad Prism software version 5.0 (La Jolla, California, USA) using paired or unpaired (depending on experiment) Student’s t-test . Depending on the experiment this was done 1-tailed or 2-tailed. Details per experiment are indicated in the corresponding figure legends. Conditions were compared to control and statistical significance was set at P < 0.05. To test for normal distribution of the input data, D’Agostino-Pearson omnibus normality tests were performed. All quantitative data sets presented here passed the normality tests. Bars in graphs represent mean ± standard deviation (SD).

## Results

### U3 snoRNA expression is decreased in cartilage and chondrocytes as function of OA

**Figure 1:**
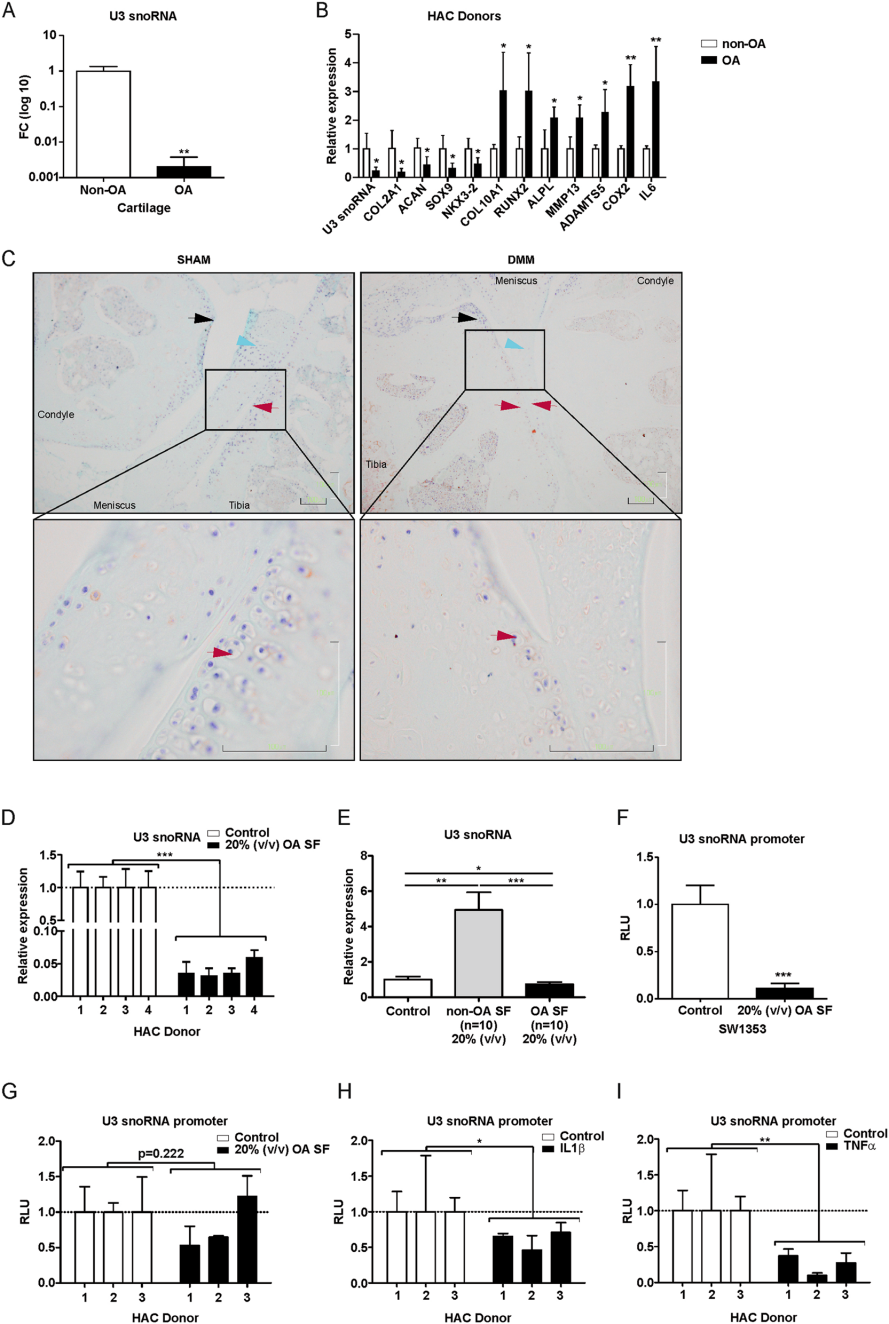
Impaired expression of U3 snoRNA in osteoarthritic cartilage and chondrocytes. (**A**) Total RNA from non-OA or OA cartilage (n = 6 donors per group) was extracted and hybridized onto Affymetrix miRNA 4.0 arrays. Using a probe set for *Homo sapiens*, differential expression of ncRNAs was determined (Supplementary Fig. 1)*.* Expression of U3 snoRNA in microarray data is depicted as Log10 fold-change. (**B**) Expression levels of U3 snoRNA; mRNA expression of chondrogenic genes (*COL2A1*; *ACAN*; *SOX9*; *NKX3-2*), hypertrophic genes (*RUNX2*; *COL10A1*, *ALPL*), matrix-degrading enzyme genes (*MMP13*, *ADAMTS5*) and inflammatory mediator genes (*COX2*; *IL6*) were determined in chondrocytes derived from OA donors relative to non-OA controls (n = 4 donors per group) using RT-qPCR analysis. Data were normalized to cyclophilin expression. (**C**) DMM (n = 3) or sham surgery (n = 3) was performed in C57BL6/J male mice. Eight weeks post-surgery mice were sacrificed and knees prepared for U3 snoRNA ISH (representative images are depicted). Bottom images show the field-of-view in the boxes from the upper images at higher magnification. Red arrow heads: load-bearing articular cartilage surfaces; black arrow heads: non-load-bearing articular cartilage surfaces; blue arrow heads: meniscus. (**D**) Non-OA chondrocytes (n = 4 donors) were cultured for 24 h in the presence or absence of OA synovial fluid (OA SF) (20% (v/v); pool of 6 donors)). Expression of U3 snoRNA was determined relative to non-treated controls by RT-qPCR analysis. Results were normalized to total DNA content in parallel wells. (**E**) Non-OA chondrocytes (n = 4 donors) were cultured for 24 h in the presence or absence of synovial fluid from non-OA (non-OA SF) or OA (OA SF) donors [20% (v/v); pool of ten donors)]. Expression of U3 snoRNA was determined relative to control condition by RT-qPCR analysis. Data were normalized to cyclophilin expression. (**F**–**I**) SW1353 cells or non-OA HACs (n = 3 donors) were transfected with a pNL1.2[NlucP]_Hs_U3_promoter plasmid. Subsequently, cells were exposed to OA synovial fluid (20% (v/v), a pool of six donors), IL1β (10 ng/ml) or TNFα (20 ng/ml) for 20 h and Nanoluc luciferase levels were measured. Data were calculated relative to control conditions (RLU). Statistical significance was determined using Student’s t-tests; (**A**/**B**/**E**–**I**) 2-tailed unpaired, (**D**) 2-tailed paired. Bars show the mean (± SD). *P < 0.05, **P < 0.01, ***P < 0.001 versus control conditions.

In order to find clues whether ribosome biogenesis is disturbed in osteoarthritic cartilage at the snoRNA level, a previously performed miRNA microarray experiment on young non-OA cartilage and old OA cartilage^19^, on which probe sets for snoRNAs were also present, was reanalyzed (Supplementary Fig. 1). A number of snoRNAs was found to be differentially expressed between non-OA and OA cartilage and amongst these U3 snoRNA expression was significantly lower in OA cartilage (Fig. 1A). Since U3 is one of the most abundant snoRNAs playing a key role in ribosome biogenesis, we investigated its involvement in chondrocyte homeostasis in more detail. To verify whether isolated OA chondrocytes have decreased U3 snoRNA levels, we measured U3 snoRNA expression in chondrocytes isolated from young non-OA and from old OA cartilage. Expression of U3 snoRNA in chondrocytes from OA cartilage was reduced (Fig. 1B) compared to non-OA chondrocytes. Reduced U3 snoRNA expression in OA chondrocytes was accompanied with a distinctive cellular phenotype associated with (pre-)hypertrophic OA chondrocytes^5,31^, with significantly reduced mRNA expression of chondrogenic genes *COL2A1*; *ACAN*; *SOX9* and *NKX3-2* and increased expression of chondrocyte hypertrophy-associated genes *COL10A1*; *RUNX2*; *ALPL*; *MMP13*; *ADAMTS5*; *COX2* and *IL6*. Since there was an age difference between the non-OA cartilage/chondrocytes and the OA cartilage/chondrocytes used to determine differences in U3 snoRNA expression (Fig. 1A/B), we next measured OA-dependent alterations in chondrocyte U3 snoRNA levels in an experimental mouse model for traumatic OA (destabilization of medial meniscus; DMM^32^). Eight weeks post-DMM surgery knees were processed for U3 snoRNA in situ hybridization (ISH). A general reduction of U3 snoRNA ISH signal was observed in articular chondrocytes within the weight-bearing area of the joint in DMM-operated compared to sham-operated knees (Fig. 1C; red arrows). This was also observed in the menisci (Fig. 1C; blue arrowheads). U3 snoRNA levels were unaltered in chondrocytes in non-weight-bearing areas of the knee joints in sham-operated *versus* DMM-operated-mice (Fig. 1C; black arrows). Next, we asked whether OA-like conditions are capable of reducing U3 snoRNA expression levels in chondrocytes. To this end, non-OA human articular chondrocytes (HACs) from different donors were exposed to 20% (v/v) OA synovial fluid. U3 snoRNA expression was significantly diminished in HACs exposed to OA synovial fluid (Fig. 1D). Exposing non-OA HACs to non-OA synovial fluid caused a significant increase in the expression of U3 snoRNA (Fig. 1E). A U3 snoRNA gene promoter-reporter assay in SW1353 cells (Fig. 1F) or in HACs (Fig. 1G) revealed that OA synovial fluid reduced U3 snoRNA promoter transcriptional activity in SW1353 cells and two out of three HAC donors. In addition we observed that U3 snoRNA promotor transcriptional activity is reduced in HACs by exposure to the two katabolic cytokines IL1β (Fig. 1H) or TNFα (Fig. 1I). Collectively, these data suggest that OA conditions are able to impact U3 snoRNA levels in chondrocytes.

### U3 snoRNA levels influence the articular chondrocyte’s transcriptomic phenotype

**Figure 2:**
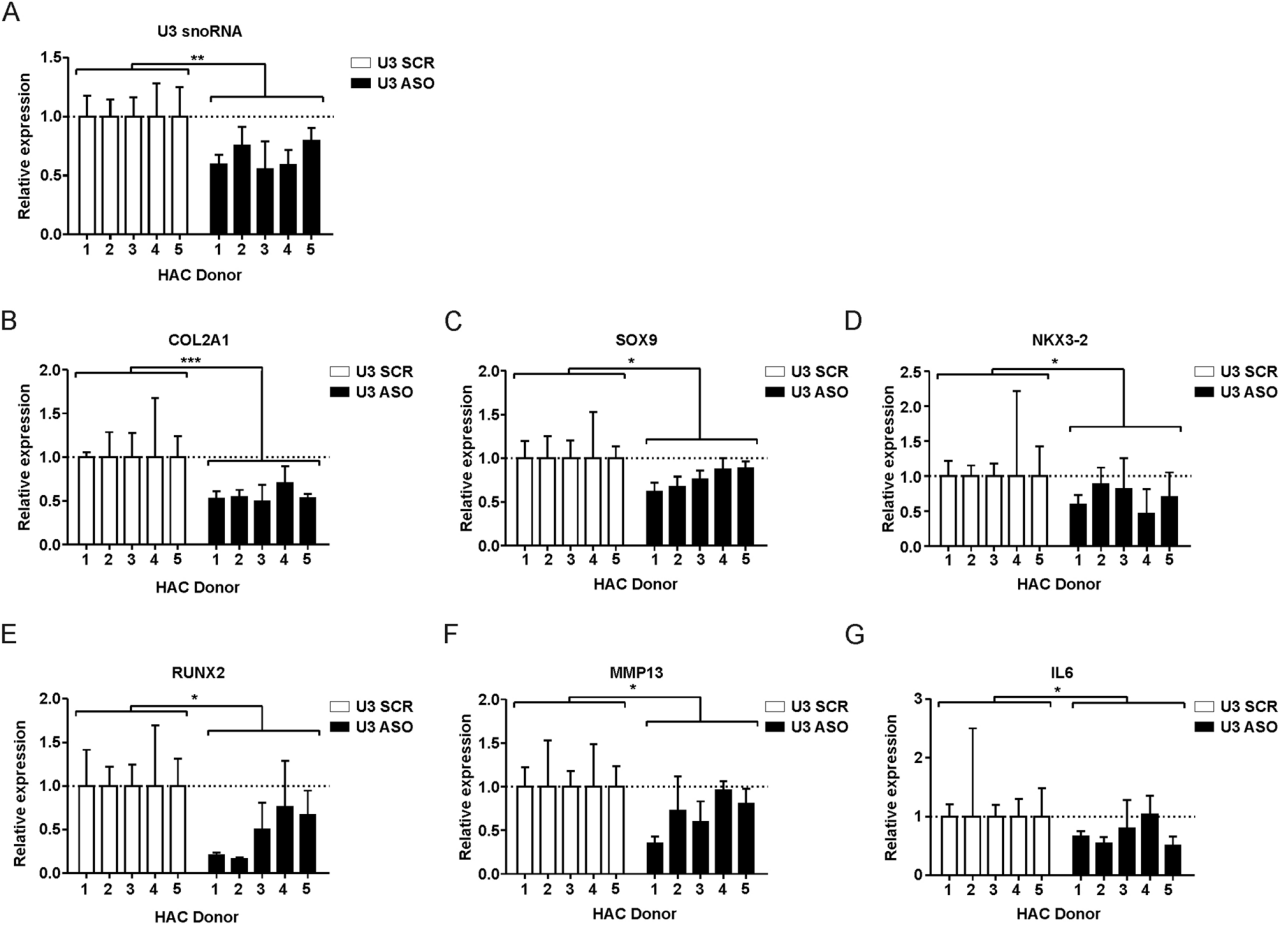
U3 snoRNA knock-down impacts the articular chondrocyte’s phenotype. Non-OA chondrocytes (n = 5 donors) were transfected with a U3 snoRNA ASO (U3 ASO) or scrambled (SCR) ASO and cultured for 24 h. (**A**) Expression of U3 snoRNA; (**B**) *COL2A1*; (**C**) *SOX9*; (**D**) *NKX3-2*; (**E**) *RUNX2*; (**F**) *MMP13* and (**G**) *IL6* mRNA levels were determined by RT-qPCR analysis. Data from U3 ASO samples were calculated relative to controls transfected with the SCR ASO. Results were normalized to relative total DNA content in parallel wells. Statistical significance was determined using 2-tailed paired Student’s t-tests. Bars show the mean (± SD). *P < 0.05, **P < 0.01, ***P < 0.001 versus control conditions.

**Figure 3:**
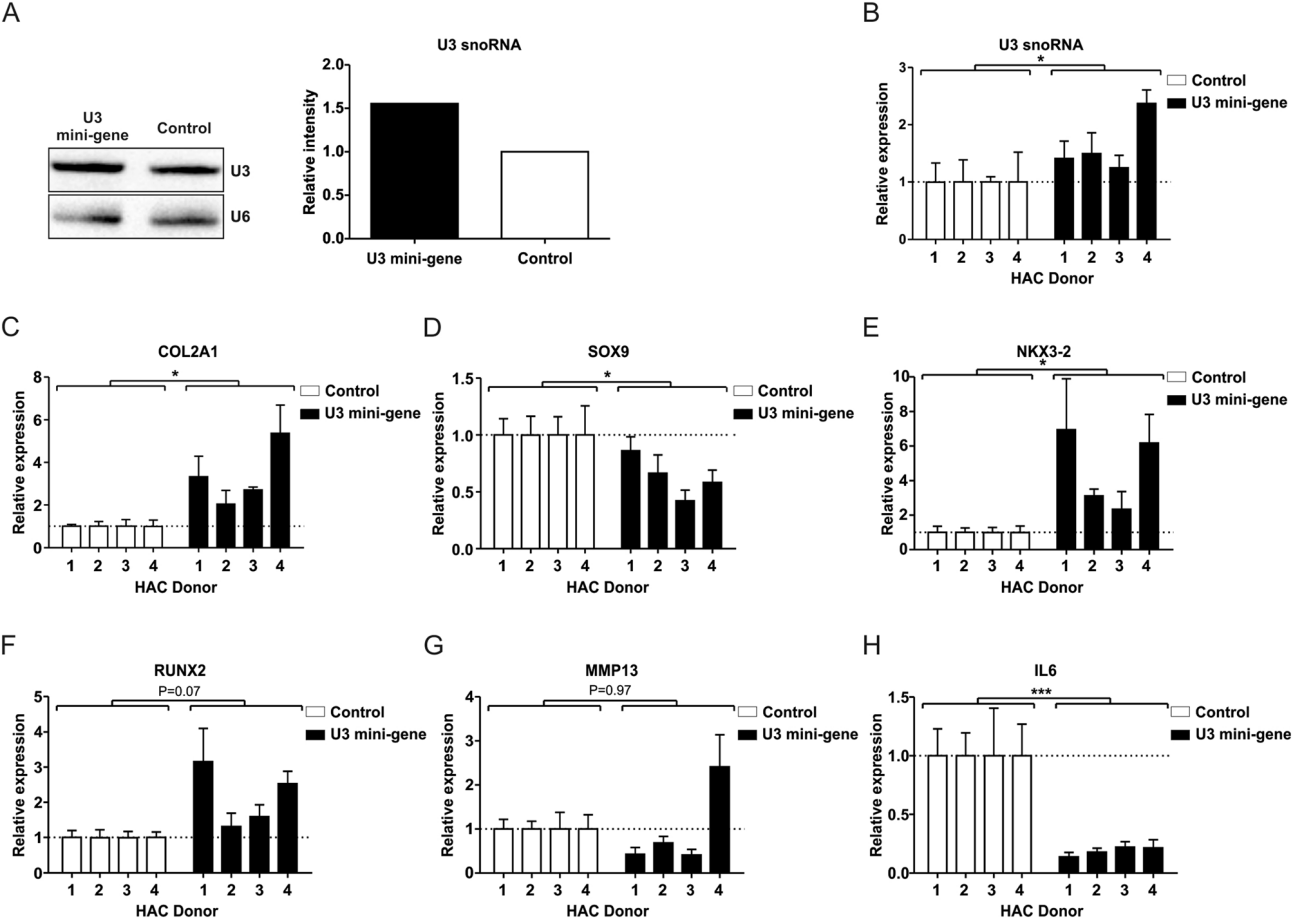
Ectopic expression of U3 snoRNA impacts the chondrocyte’s phenotype. (**A**) Activity of the U3 mini-gene in primary chondrocytes was confirmed by northern blot (n = 1) left panel. U3 snoRNA expression levels were calculated relative to control and normalized for U6 snRNA, right panel. Non-OA chondrocytes (n = 4 donors) were transfected with the U3 mini-gene (10 ng plasmid/cm^2^) and cultured for 24 h. (**B**) Expression levels of U3 snoRNA; (**C**) *COL2A*; (**D**) *SOX9*; (**E**) *NKX3-2*; (**F**) *RUNX2*; (**G**) *MMP13* and (**H**) *IL6* gene expression levels were determined relative to control. Results were normalized to total DNA content in parallel wells. Statistical significance was determined using Student’s t-tests; (**B**) 1-tailed paired, (**C**–**H**) 2-tailed paired. Bars show the mean (± SD). *P < 0.05, **P < 0.01, ***P < 0.001 versus control conditions.

It is unknown whether the level of U3 snoRNA expression influences the chondrocyte phenotype on the transcriptomic level. Therefore we altered U3 snoRNA expression levels in non-OA primary chondrocytes using a U3 snoRNA-specific antisense oligonucleotide (ASO). Following ASO transfections in five individual non-OA chondrocyte cultures, reduced U3 snoRNA expression was confirmed (Fig. 2A). As a result, expression of *COL2A1*, *SOX9*, *NKX3-2*, *RUNX2*, *MMP13* and *IL6* was significantly reduced in all five chondrocyte isolates (Fig. 2B–G), but with variable levels of change. Reciprocally, we ectopically increased expression of U3 snoRNA moderately by transfecting primary chondrocytes with a U3 mini-gene (Fig. 3). The activity of the U3 mini-gene was confirmed by northern blot (Fig. 3A) and significantly elevated U3 snoRNA expression was confirmed in primary chondrocytes (Fig. 3B). The elevated U3 snoRNA expression levels resulted in upregulated levels of mRNAs coding for *COL2A1* and *NKX3-2* (Fig. 3C/E), while expression of *SOX9* and *IL6* transcripts was reduced (Fig. 3D/H). Expression of *RUNX2* and *MMP13* was also altered following ectopic expression of U3 snoRNA, albeit with strong inter-donor variability (Fig. 3F/G). Taken together, we demonstrated that ectopically-induced alterations in the expression levels of U3 snoRNA in articular chondrocytes change the chondrocyte’s transcriptomic phenotype.

### Chondrocyte rRNA levels are reduced in OA conditions and are regulated by U3 snoRNA

**Figure 4:**
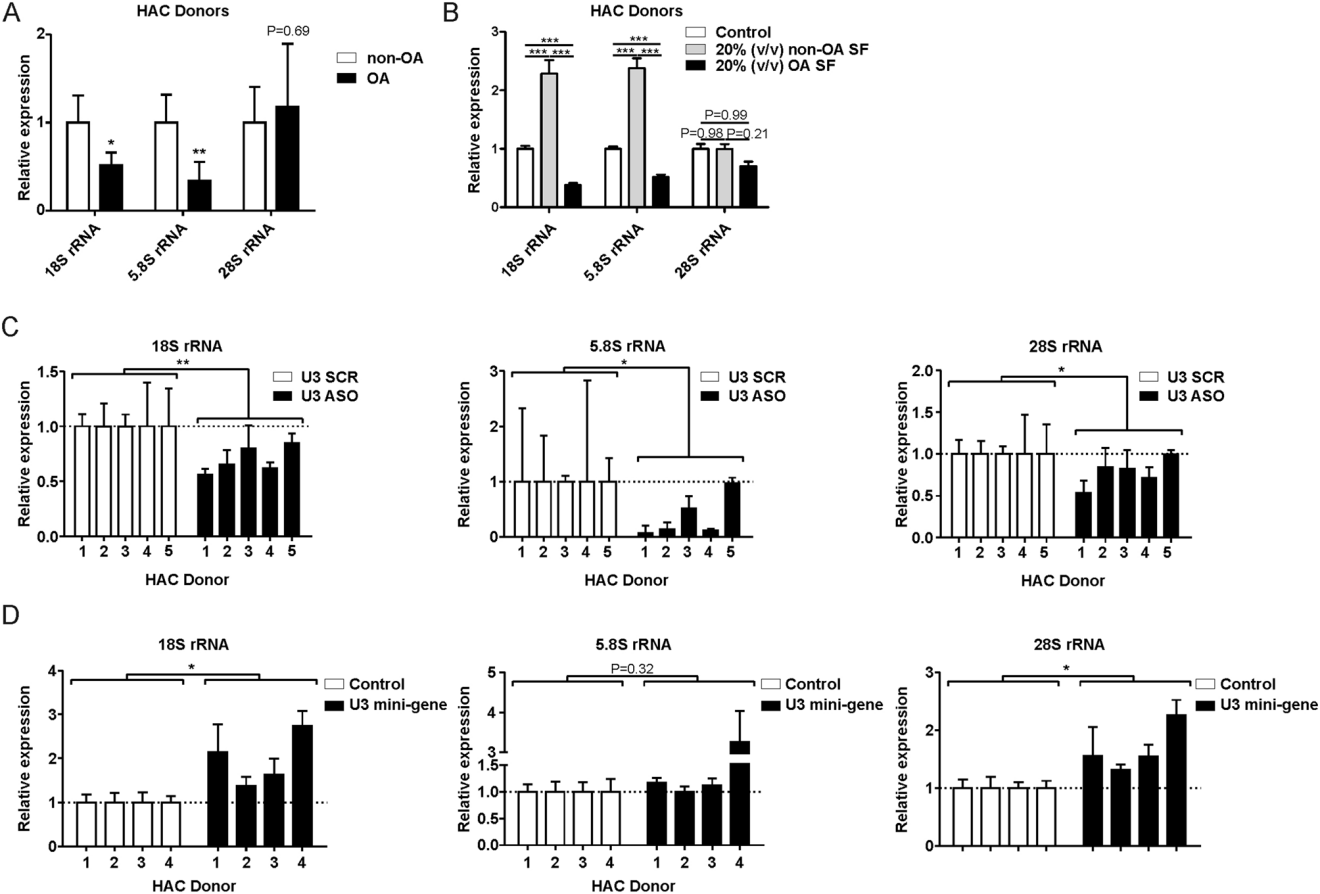
Chondrocyte ribosomal RNA expression is impacted in OA conditions and following alteration of U3 snoRNA levels. (**A**) rRNA expression levels were determined in OA and non-OA primary chondrocytes (n = 4 donors per group). (**B**) Non-OA chondrocytes (n = 4 donors) were cultured for 24 h in the presence or absence of synovial fluid from non-OA or OA donors [20% (v/v); pool of ten donors)]. Expression of rRNAs was determined relative to control condition by RT-qPCR analysis. Data were normalized to cyclophilin expression. (**C**) Non-OA chondrocytes (n = 5 donors) were transfected with a U3 snoRNA ASO or SCR ASO and cultured for 24 h and rRNA levels were measured using RT-qPCR. (**D**) Non-OA chondrocytes (n = 4 donors) were transfected with a U3 mini-gene (10 ng plasmid/cm^2^) and cultured for 24 h and rRNA levels were determined by RT-qPCR. Data were normalized to cyclophilin (**A**/**B**) or to relative total DNA content in parallel wells (**C**/**D**). Statistical significance was determined using Student’s t-tests; (**A**/**B**) 2-tailed unpaired, (**C**/**D**) 2-tailed paired. Bars show the mean (± SD). *P < 0.05, **P < 0.01, ***P < 0.001 versus control conditions.

U3 snoRNA is rate-limiting in the generation of mature rRNAs^12,33^ and 18S rRNA in particular. To determine whether rRNA levels are altered in primary chondrocytes in OA conditions, we measured 18S, 5.8S and 28S rRNA expression. In concert with reduced U3 snoRNA levels in old OA chondrocytes (Fig. 1A/B), we observed reduced expression levels of 18S and 5.8S rRNAs in old OA chondrocytes, while 28S rRNA levels remained unaltered (Fig. 4A). Following the exposure of HACs to OA synovial fluid we observed a reduction of 18S and 5.8S transcript levels (Fig. 4B), while 28S rRNA expression was not significantly altered. OA synovial fluid also reduced expression of 18S and 5.8S rRNAs in HACs (Fig. 4B), while an opposite response of 18S and 5.8S rRNA levels was detected when HACs were exposed to non-OA synovial fluid (Fig. 4B). In both conditions 28S rRNA expression was not significantly changed. Alteration of U3 snoRNA expression in primary chondrocytes (Figs. 2 and 3) led to changes in chondrocyte rRNA levels. Reduction of U3 led to reduced 18S and 28S rRNA levels, with a sharp decrease of 5.8S rRNA levels in four out of five chondrocyte donors (Fig. 4C). Ectopic U3 expression led to increased 18S and 28S rRNA levels (Fig. 4D), while not changing 5.8S rRNA levels in three out of four chondrocyte donors. Overall, data demonstrate that 18S and 5.8S rRNA levels in chondrocytes are susceptible to OA conditions and rRNA transcript levels respond to U3 snoRNA expression.

### U3 snoRNA levels influence the activity of the chondrocyte protein translation apparatus

**Figure 5:**
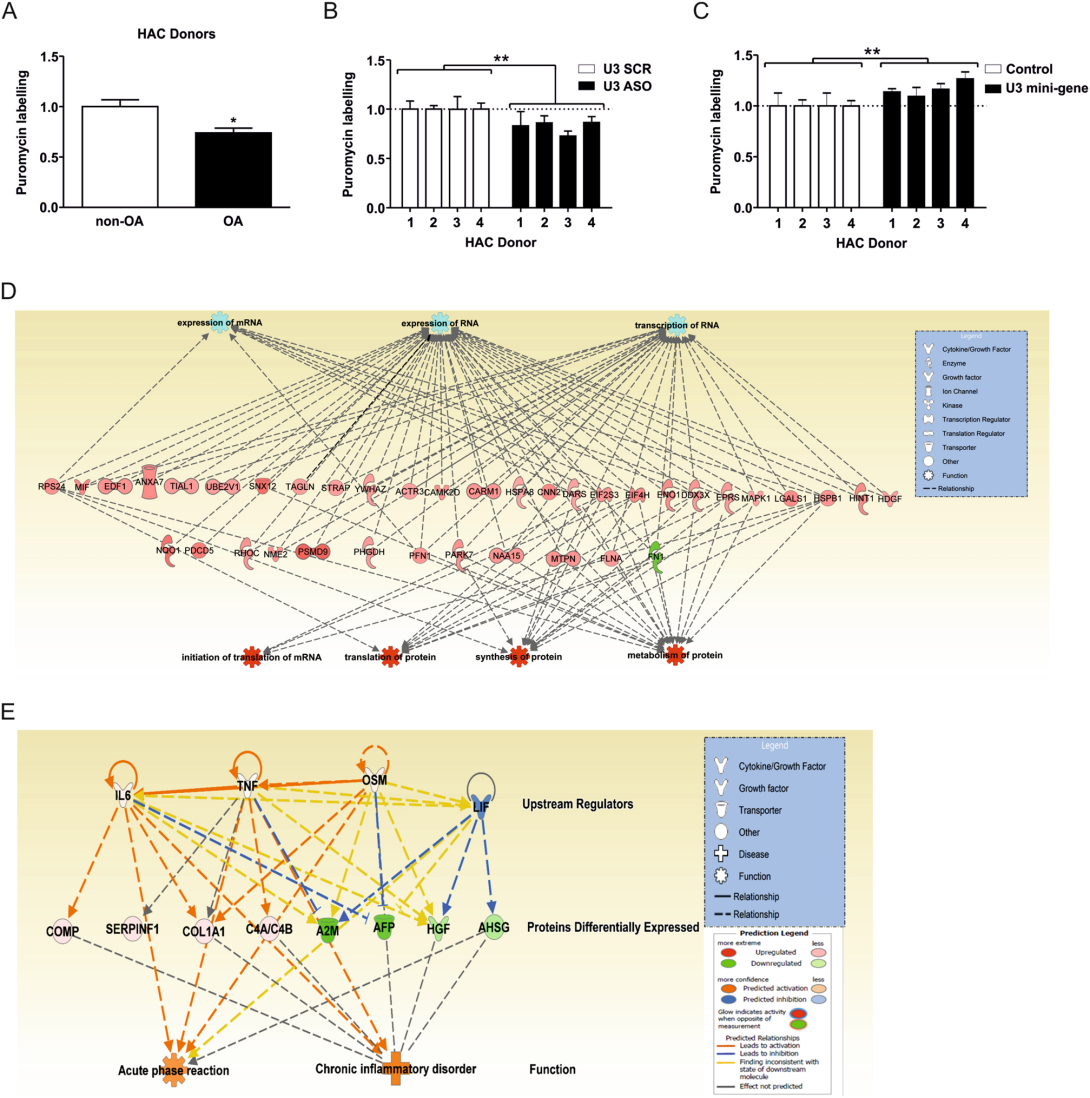
U3 snoRNA expression levels influence the chondrocyte protein translation capacity and its proteome. Protein translational capacity was determined using a puromycilation assay in a number of conditions: (**A**) OA or non-OA primary chondrocytes (n = 4 donors per group), (**B**) in U3 snoRNA knockdown conditions in non-OA HACs (n = 4 donors) transfected with a U3 snoRNA ASO or SCR ASO, (**C**) and in non-OA primary chondrocytes (n = 4 donors) transfected with a U3 mini-gene (10 ng plasmid/cm^2^). Puromycilation data (**A**–**C**) were calculated relative to corresponding controls and were normalized to DNA content. Statistical significance was determined using Student’s t-tests; (**A**) 2-tailed unpaired, (**B**/**C**) 2-tailed paired. Bars show the mean (± SD). *P < 0.05, **P < 0.01, ***P < 0.001 versus control conditions. (**D**) SW1353 cells were transfected with a U3 ASO or SCR control and cultured for 24 h (knockdown was confirmed in Supplementary Fig. 2), after which LC–MS/MS and label-free quantification was conducted. Ingenuity pathway analysis was performed on the differentially expressed proteins between conditions. Green nodes; decreased expression following knockdown, red nodes; increased expression following knockdown. Intensity of colour is related to a higher fold-change. Key to the main features in the networks is shown. (**E**) Upstream analysis in Ingenuity Pathway Analysis on the differentially expressed secreted proteins between conditions [(**D**) U3 snoRNA knockdown in SW1353]. This analyses linkage to differentially expressed proteins through coordinated expression, identify potential upstream regulators that has been observed experimentally to affect gene expression. Green nodes; decreased expression following U3 snoRNA knockdown, red nodes; increased expression following knockdown. Intensity of colour is related to a higher fold-change. Key to the main features in the networks is shown.

Our data demonstrate alterations in chondrocyte rRNA levels in OA conditions and following knock-down or ectopic expression of U3 snoRNA. Since rRNAs are the catalytically active subunits of the translating ribosome, we next investigated the consequences for overall protein translational capacity. Protein translational capacity was determined in chondrocytes from young non-OA and old OA cartilage and a significantly reduced translational capacity was observed in osteoarthritic chondrocytes (Fig. 5A). Translational capacity of chondrocyte cultures with diminished U3 snoRNA expression was significantly reduced (Fig. 5B), while ectopic induction of U3 snoRNA expression resulted in a mild, but significantly increased translational capacity (Fig. 5C). These data show that U3 snoRNA is able to change the chondrocyte’s translational capacity. To determine the full impact of U3 snoRNA knockdown and the concomitant reduction in rRNA levels on the chondrocyte translational apparatus, we subsequently conducted whole proteome analysis on SW1353 cells in which U3 snoRNA expression was reduced. A reduction of U3 snoRNA and concomitant reduction in 18S and 5.8S rRNA levels were confirmed (Supplementary Fig. 2). Ingenuity Pathway Analysis (IPA) analysis of the differential proteomes revealed that IPA networks “synthesis of protein”, “translation of protein”, “metabolism of protein” and “initiation of translation of mRNA” were top deregulated networks (Fig. 5D) and confirm a global impact of the reduction of U3 snoRNA expression on protein translational processes. Also networks involving transcription and expression of RNA were prominently represented in the IPA analysis. The differential proteome that is observed following U3 snoRNA reduction concerns a particular global upregulation of differentially expressed proteins (Supplementary Fig. 3), rather than a downregulation and contains a great number of protein species involved in the process of protein translation and ribosomal stress responses. An additional proteomic analysis of the secretome of these U3 snoRNA knockdown cells was conducted. Relevant to inflammatory responses in OA cartilage, we could demonstrate that secreted proteins involved in antagonizing inflammatory processes were present in the secretomes of U3 knockdown cells in lower abundance (Fig. 5E). Together these data show alterations in chondrocyte protein translational activity following alterations in U3 snoRNA expression levels, with U3 snoRNA impacting protein translation molecular routes.

### U3 snoRNA expression is increased by BMP7

**Figure 6:**
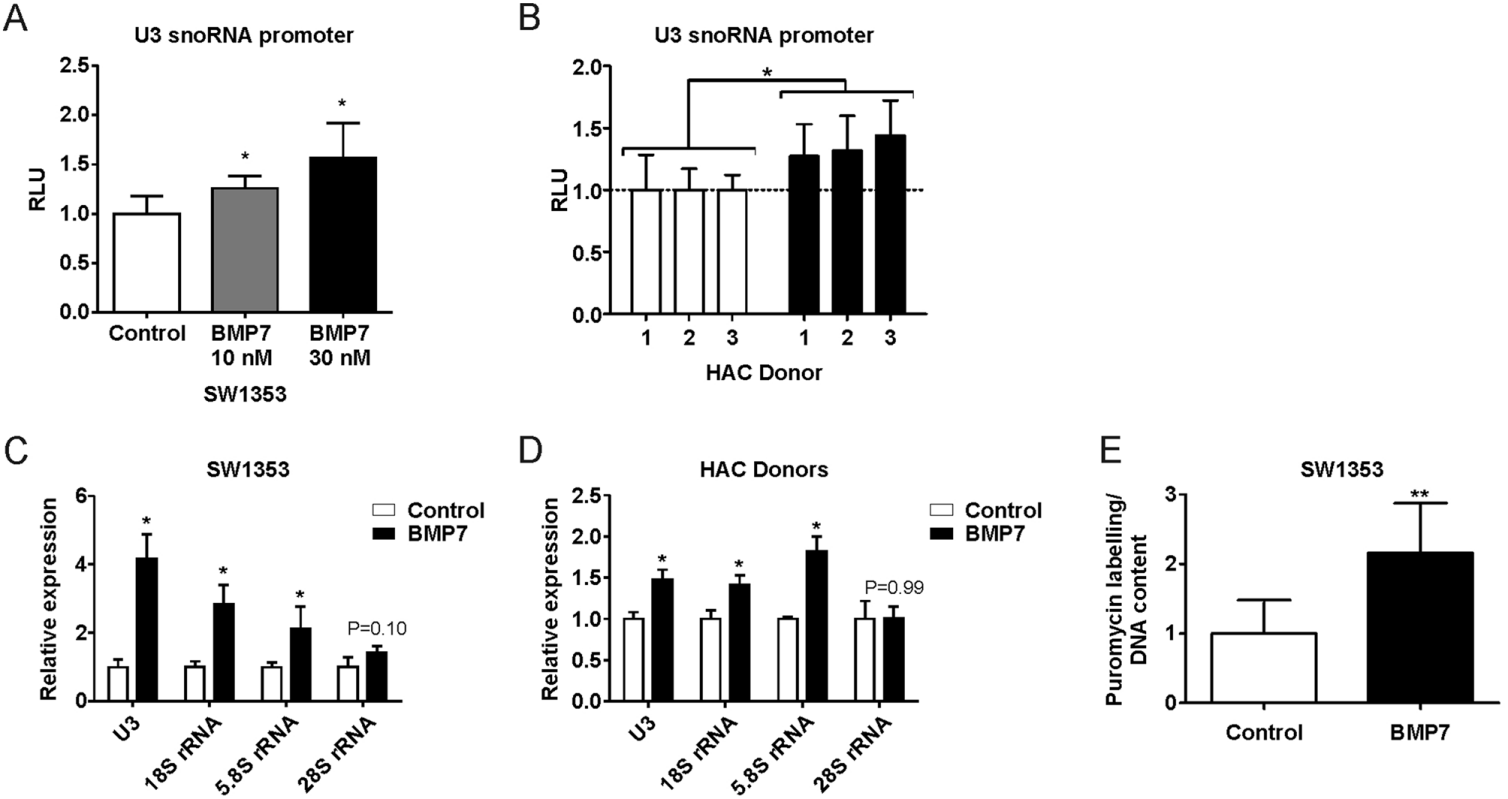
U3 snoRNA and rRNA expression levels are regulated by BMP7. (**A**) SW1353 cells or (**B**) non-OA primary chondrocytes (n = 3) were transfected with a pNL1.2[NlucP]_Hs_U3_promoter plasmid. Subsequently, SW1353 cells (**A**) were exposed to 10 or 30 nM BMP7 and HACs (**B**) to 10 nM BMP7 for 20 h and Nanoluc luciferase levels were measured. Data were normalized and calculated relative to control conditions (RLU). (**C**) SW1353 cells and (**D**) non-OA primary chondrocytes (n = 4), were exposed to 1 nM BMP7 for 24 h. Expression of U3 snoRNA; 18S, 5.8S and 28S rRNA was determined relative to control conditions by RT-qPCR analysis. Results were normalized to cyclophilin expression. (**E**) SW1353 cells were exposed to 1 nM BMP7 for 24 h. Protein translation capacity was determined using a puromycilation assay and data were normalized for DNA content. Statistical significance was determined using Student’s t-tests; (**A**/**B**) 1-tailed unpaired, (**C**/**E**) 2-tailed unpaired, (**D**) 2-tailed paired. Bars show the mean (± SD). *P < 0.05, **P < 0.01, ***P < 0.001 versus control conditions.

We previously demonstrated that bone morphogenetic protein 7 (BMP7) is capable of ameliorating the OA chondrocyte phenotype^5^. Considering this anabolic action of BMP7 we evaluated whether U3 snoRNA and its subsequent rRNA targets can be induced in chondrocytes by BMP7. A U3 snoRNA promoter-reporter experiment in SW1353 cells or in non-OA HACs demonstrated that U3 snoRNA promoter transcriptional activity was induced following stimulation with BMP7 (Fig. 6A/B). In both chondrocyte cell models BMP7 induced U3 gene expression and increased expression of 18S and 5.8S rRNAs (Fig. 6C/D). The expression of 28S rRNA was not significantly altered. SW1353 cells stimulated with BMP7 had an increased translational capacity (Fig. 6E), supporting the notion that the BMP7-mediated induction of U3 snoRNA and rRNA expression increases the chondrocyte’s translational capacity.

## Discussion

U3 snoRNA is an abundant snoRNA with a pivotal function in the generation of mature 18S rRNA in eukaryotes^14^. It’s snoRNP biochemistry^34^ and mechanism-of-action^35^ have been extensively studied in the past decades. However, it was only recently described to be univocal involved in 18S rRNA generation in human cells^12^. In contrast with its decade-long thorough molecular characterization, insight into its involvement in human disease is limited to a role in cell proliferation and tumorigenesis^12,36^, while it remained unknown whether U3 snoRNA levels can be regulated via extracellular cues.

We demonstrate that in different osteoarthritic conditions the expression level of U3 snoRNA in articular chondrocytes is diminished. Reduced expression of U3 snoRNA was previously reported in serum of ageing mice^37^, and as a result of nucleus pulposus ageing in mice^38^. As a general study limitation in this field, we could not investigate the potential influence of age difference on U3 snoRNA expression between non-OA and OA cartilage/chondrocytes. To circumvent potential U3 expression interpretation issues, we reinforced our analyses by performing additional U3 snoRNA expression analyses in a murine model for OA and used synovial fluid from OA and non-OA individuals (no statistically significant age difference) on chondrocytes. Both as models in which age was not a potential confounder. Data from these models confirmed the OA-dependent reduction of chondrocyte U3 snoRNA expression. We could also conclude that reduced OA chondrocyte U3 snoRNA expression is, at least in part, mediated via diminished U3 gene transcription activity. This is the first data demonstrating that the extracellular environment is capable of controlling cellular U3 snoRNA levels, thereby tuning the cell’s capacity to generate mature rRNA species. U3 snoRNA is transcribed from a dedicated transcriptional unit under the control of an RNA polymerase II-driven (RNAPII) promoter. Little is known about the control of snoRNA transcription from dedicated transcriptional units, but our group previously reported that transcriptional activity of another dedicated snoRNA gene (RMRP) can also be influenced via major chondrocyte signaling pathways during chondrogenesis^39^. OA synovial fluid is a complex body fluid containing a plethora of catabolic morphogens and cytokines/chemokines^40,41^. Therefore we expect that the signaling molecule composition of OA synovial fluid acts on major chondrocyte signaling pathways, leading to the observed decreased U3 snoRNA expression. Indeed our data demonstrated that the katabolic cytokines IL1β and TNFα attenuated U3 promoter activity. Reciprocally, we demonstrate that non-OA synovial fluid and BMP7 are capable of inducing U3 snoRNA transcription and subsequent U3 snoRNA levels in chondrocytes.

A network of snoRNAs is involved in key ribosome biogenesis processes^16^, and U3 snoRNA is a factor in the generation of 18S rRNA^14^. Indeed our data show that manipulation of chondrocyte U3 snoRNA levels impacts 18S rRNA levels, but also alters 5.8S and 28S rRNA levels in many instances. Taking into consideration the 47S pre-rRNA multi-cistronic origin of 18S rRNA, we speculate that aberrations in 18S rRNA levels may impact 5.8S and 28S rRNA levels possibly due to a 40S over 60S ribosomal subunit imbalance^42^. Also other snoRNAs involved in 47S pre-rRNA processing could be impacted by OA conditions. Our unpublished data suggest deregulated chondrocyte U13 and RMRP snoRNA levels in OA conditions. This may provide additional explanations for the observed abberations in the levels of other rRNAs. In contrast with our current findings, other recent work demonstrated increased protein translation activity in OA cartilage^43–45^. The OA cartilage used in our study was derived from end-stage (K&L grade 3–4) knee OA patients, while the OA grade of the human OA-lesioned cartilage presented by others^45^ appears moderate. Taking into account that the reported increased protein translation activity in a rat model for OA seems to fade with progression into late OA development^45^, we speculate that the contradictory observations may be explained by OA severity of the analyzed cartilage. This is further supported by the idea that an inflammatory component is much more dominant in early OA than in end-stage OA^46^, and it was shown that the increase in translation activity could be recapitulated by IL1β^45^. This suggests that an increase in chondrocyte translation activity in OA may be predominantly related to early OA, while impaired protein translation may be a hallmark of end-stage OA.

While increased chondrocyte protein synthesis capacity in early OA may be related to mTOR signaling and imbalanced autophagy^47^, it is unknown what the specific consequence is of a diminished capacity of global protein synthesis in relation to development or progression of osteoarthritis. We propose that U3 snoRNA-related reduction of chondrocyte protein translation affects the chondrocyte’s ability to maintain the molecular build-up of the cartilage proteinaceous ECM. Alterations in protein translation capacity may render articular cartilage vulnerable to any biomechanical, catabolic, traumatic or age-related changes in the joint that are associated with OA development and progression^2^. In addition, our gene expression data, which are a representation of the overall chondrocyte differentiation status^3,4^, show that alterations in chondrocyte U3 snoRNA levels affects its differentiation status. Data from the secretome proteomics analysis further show U3-dependent alterations in the levels of secreted proteins involved in balancing inflammatory pathways (A2M; AFP; HGF; AHSG). Together this shows that besides an influence on protein translation, alterations in chondrocyte U3 snoRNA levels additionally impact the chondrocyte’s homeostatic integrity.

Our whole proteome analysis uncovered that reduction of U3 snoRNA levels induces ribosome stress, which is indicated by aberrations in molecular pathways controlling key aspects of rRNA and ribosomal protein gene transcription (PGAM1; EDF1; MAPK1; AHCY; CARM1; FLNA), rRNA processing (FSCN1; RPS8; RAN; RPS24), ribosomal subunit transport (RAN), translation initiation (PHGHD; AKT1S1; EIF4H; ARPC5; TIAL1; EIF2S3; PABPC1; HSPB1; TPT1; FN1), chain elongation (DARS; EPRS; DDX3X) and protein folding/chaperoning (AHSA1; HSPA8; HSP90; HSPB1) (Supplementary Fig. 3). Ribosome stress following reduction of U3 snoRNA expression was previously reported and is accompanied by stabilization of p53^12^. Indeed we also observed increased expression of p53 after U3 snoRNA knockdown in SW1353 cells (data not shown). It was previously reported that p53 expression is also increased in OA chondrocytes^48^. Stabilisation of p53 feeds back to ribosome biogenesis and down regulates translational activity^49,50^. Besides the U3 snoRNA-dependent availability of rRNAs for ribosome biogenesis, this may provide a link between our observed OA-related impairment of U3 snoRNA expression levels in chondrocytes and their translational activity. In addition, IPA analyses of the U3 knock-down cellular proteome demonstrated that a great number of the differentially expressed proteins are targets of Myc (data not shown). We therefore speculate that stabilization of p53 may impact chondrocyte gene transcription processes via Myc, providing a potential explanation for the impact and discrepancies found in chondrocyte gene expression following alteration of U3 levels (Figs. 2, 3).

To comprehend the implications of the larger snoRNA network^16^ on the function of the articular chondrocyte, other members of the non-canonical and canonical classes of snoRNAs should also be investigated. However, our study for the first time demonstrates the involvement of a snoRNA in articular chondrocyte biology. The OA-related impaired expression of U3 snoRNA has detectable consequences for articular chondrocyte protein translation capacity, rendering U3 snoRNA expression a potential therapeutic target in OA treatment. Indeed our data show that U3 snoRNA expression can be induced by the growth factor BMP7, providing a potential manner to target U3 snoRNA in OA chondrocytes.

## Supplementary information

Supplementary Information.
