# Supplementary material for: Impaired chondrocyte U3 snoRNA expression in osteoarthritis impacts the chondrocyte protein translation apparatus
Source: Sci Rep. 2020 Aug 10;10:13426. doi: 10.1038/s41598-020-70453-9 (PMC7417995; doi:10.1038/s41598-020-70453-9)
Supplement: Supplementary file 1 — Supplementary Information. [file 41598_2020_70453_MOESM1_ESM.docx]

**SUPPLEMENTARY FILES TO**

**Impaired chondrocyte U3 snoRNA expression in osteoarthritis impacts the chondrocyte protein translation apparatus**

E.G.J. Ripmeester^1^, M.M.J. Caron^1^, G.G.H. van den Akker^1^, D.A.M. Surtel^1^, A. Cremers^1^, P. Balaskas^3^, P. Dyer^3^, B.A.C. Housmans^1^, A. Chabronova^1^, A. Smagul^3^, Y Fang^3^, L.W. van Rhijn^1,2^, M.J. Peffers^3^*, T.J.M. Welting^1,2^*

^1^Laboratory for Experimental Orthopedics, Department of Orthopedic Surgery, Maastricht University, Universiteitssingel 50, 6229 ER, Maastricht, the Netherlands.

^2^Laboratory for Experimental Orthopedics, Department of Orthopedic Surgery, Maastricht University Medical Center. P.O. Box 5800, 6202 AZ, Maastricht, the Netherlands.

^3^Department of Musculoskeletal Biology, Institute of Ageing and Chronic Disease, University of Liverpool, Liverpool L69 3BX, United Kingdom.

*= equally contributed

**
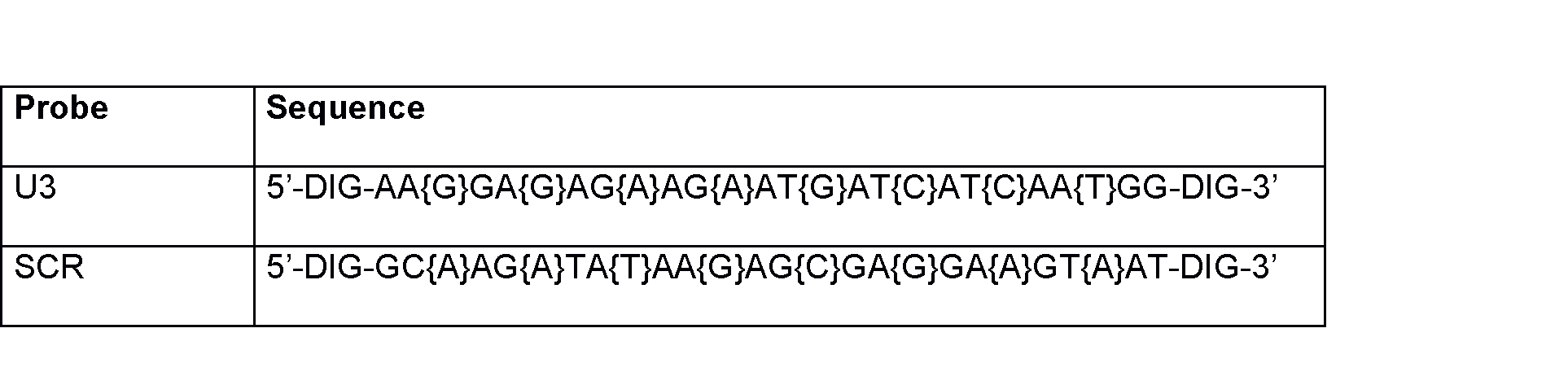
**

**Supplementary Table 1: *In situ* hydridisation probe sequences**

Digoxigenin-labelled DNA probe sequences for U3 snoRNA and Scrambled (SCR). Nucleotides between {} are LNA nucleotides.

**
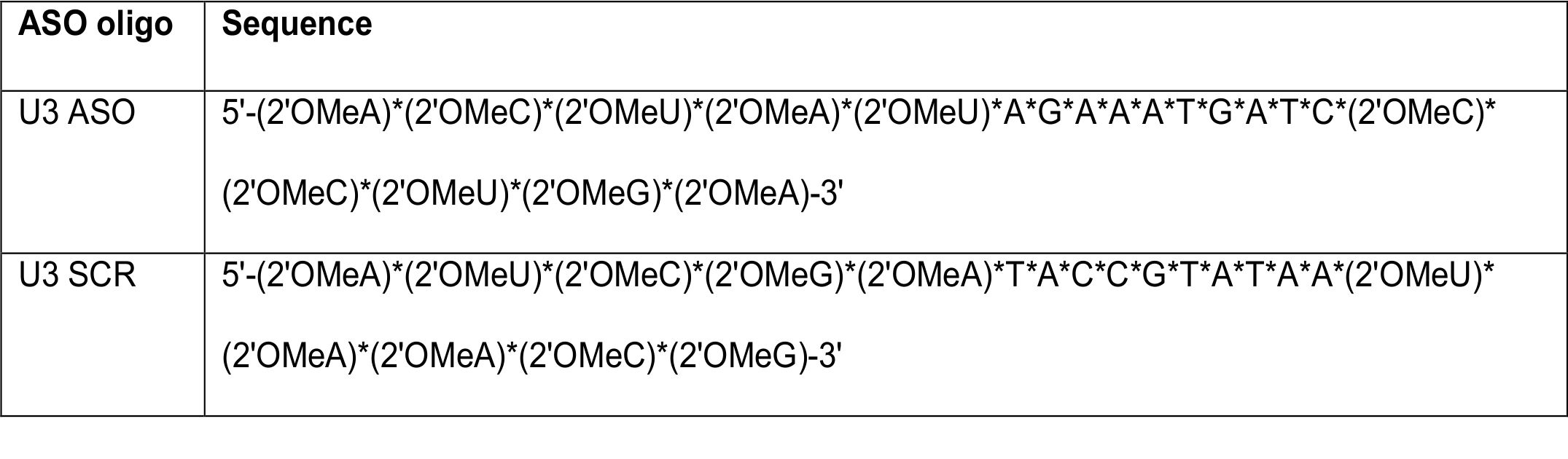
Supplementary Table 2: Antisense oligonucleotide sequences**

U3 snoRNA (U3 ASO) and scrambled (U3 SCR) ASO sequences. RNA nucleotides are indicated as between brackets, 2’OMe indicates a 2’-*O*-ribose methylated nucleotide, * indicates phosphorothioate backbone linkage.

**
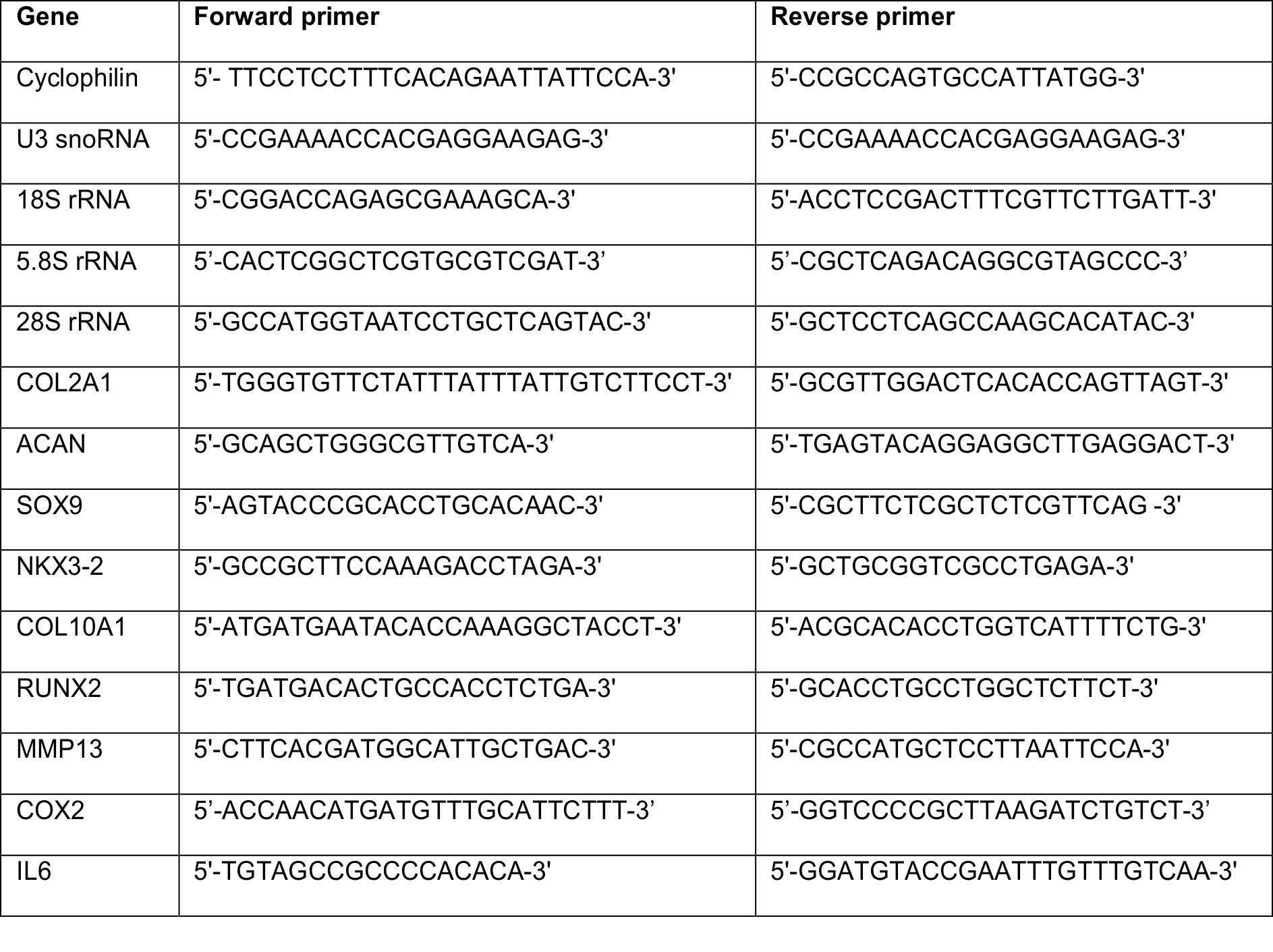
Supplementary Table 3: DNA oligonucleotide sequences for RT-qPCR**

Forward and reverse DNA oligonucleotide sequences used as primers in RT-qPCR analyses.


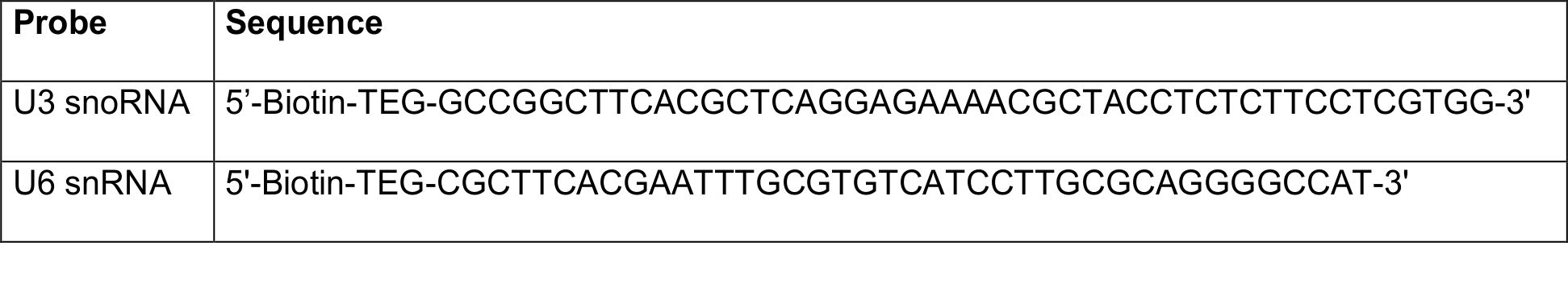
**Supplementary Table 4: Northern blot probe sequences**

Probe sequences for U3 snoRNA and U6 snRNA with 5’ biotin labels and TEG (triethyleneglycol) spacer.


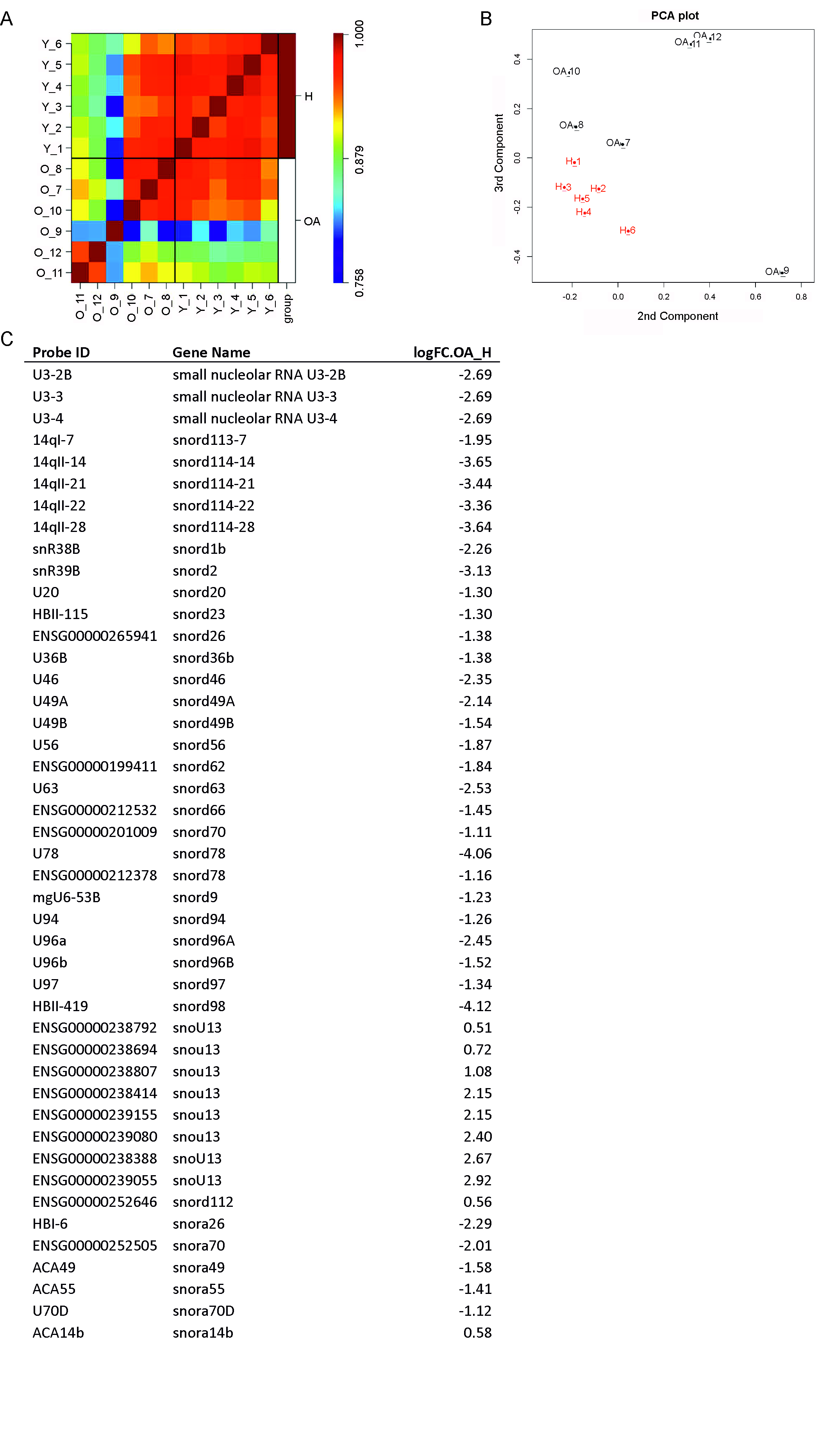


**Supplementary Figure 1: Microarray snoRNA data.**

Visualisation of data from Affymetrix GeneChip miRNA 4.0 microarray analysis for 12 samples from Young (n=6 individual donors) and OA cartilage (n=6 individual donors)[^1^](#_ENREF_1). **(A)** Heat map of hierarchical clusters of correlations among samples. Pearson’s correlation coefficients were computed using logarithm transformed snoRNA expression data from all snoRNA probes that were detected. **(B)** A 2D PCA plot of the second and third components from PCA of logarithm-transformed snoRNA abundance data. **(C)** Differentially expressed snoRNAs in these microarray data. FDR adjusted p value <0.05. Abbreviations; Y or H is Young Healthy cartilage, O or OA is OA cartilage.

**
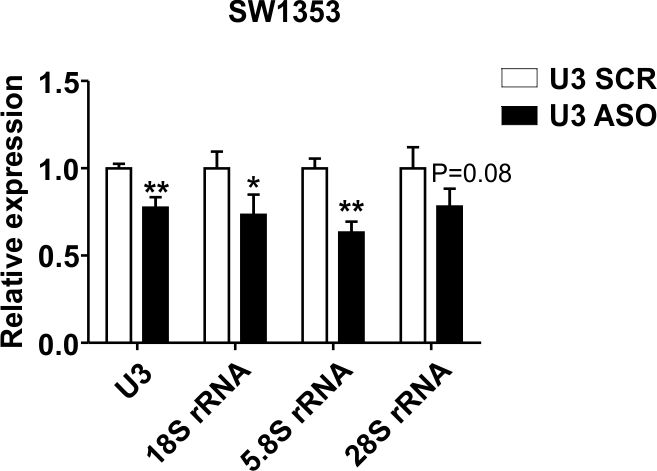
**

**Supplementary Figure 2: Knockdown of U3 snoRNA and reduction of rRNA levels in SW1353 cells for protein mass spectrometry analysis**.

In parallel with samples for proteomic analysis, reduction of U3 snoRNA expression was confirmed by RT-qPCR following transfection of U3 snoRNA ASO *versus* SCR ASO. Subsequent changes in 18S, 5.8S and 28S rRNA levels were determined by RT-qPCR. Data were normalized to cyclophilin levels and changes were calculated relative to SCR conditions by RT-qPCR analysis. Statistical significance was determined using 2-tailed unpaired Student’s t-test. Bars show the mean (±SD). * P<0.05, ** P<0.01, *** P<0.001 versus control conditions.

**
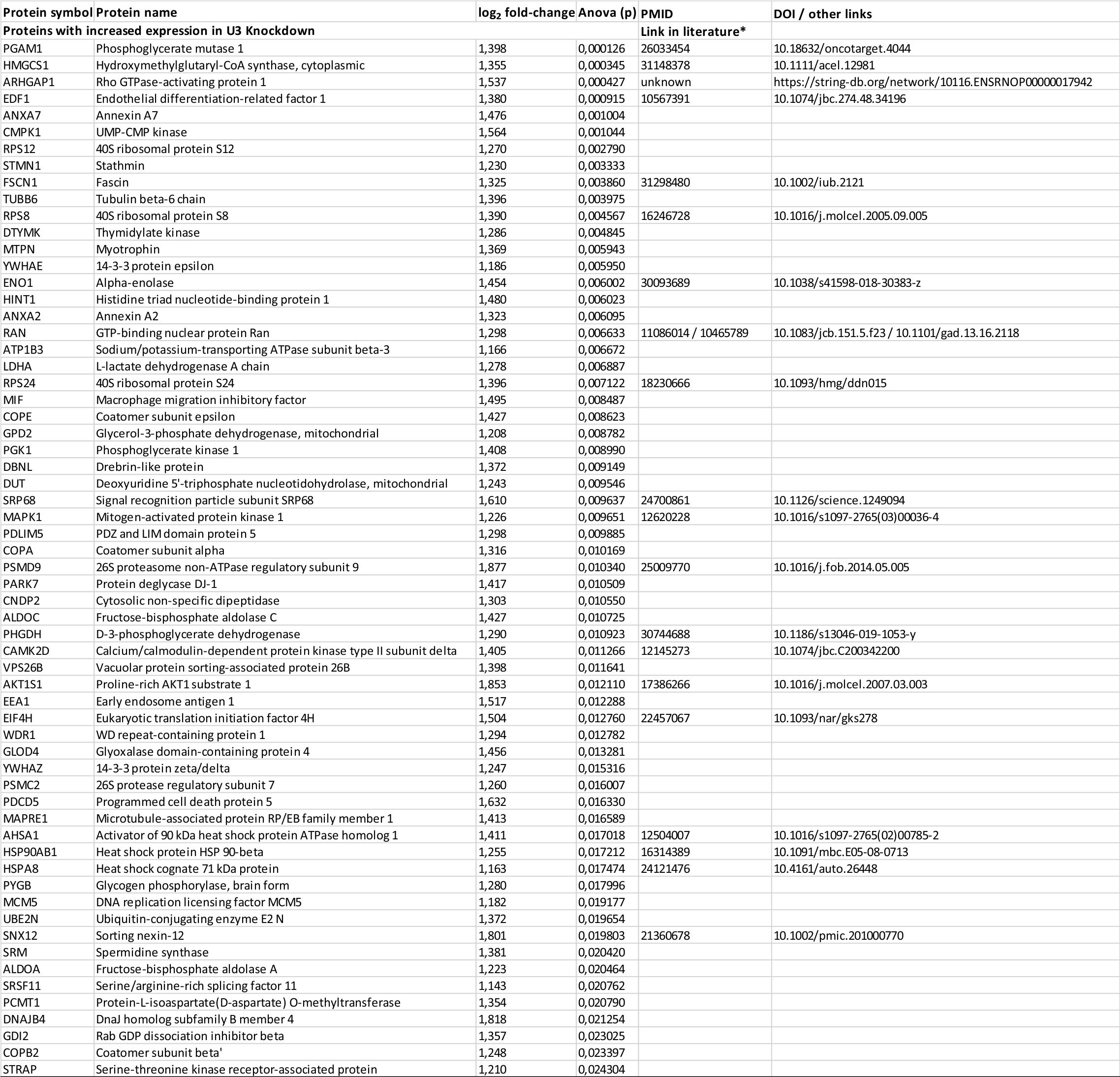
Supplementary Figure 3:** To determine the full impact of reduction of U3 snoRNA levels in the chondrocyte’s proteome, LC-MS/MS with label-free quantification was performed on the cellular proteomes of SW1353 cells transfected for 24 hours with U3 snoRNA ASO or SCR ASO. Progenesis QI software (version 4, Waters, Manchester, UK) was used to identify fold changes in protein abundance. Only unique peptides were used for quantification, and with p-values <0.05, were considered to be differentially expressed. Statistical analysis was performed using Progenesis QI software; briefly transformed normalised abundances were used for one-way analysis of variance (ANOVA) and all peptides (with p < 0.05) of an identified protein were included. Differentially expressed proteins are listed. This list was used to perform the IPA analysis shown in Figure 5D. Identified references to literature that connects the differentially expressed proteins to transcription, rRNA processing, ribosomal subunit (transport), translation initiation, chain elongation and protein folding/chaperone are provided in the list.

**
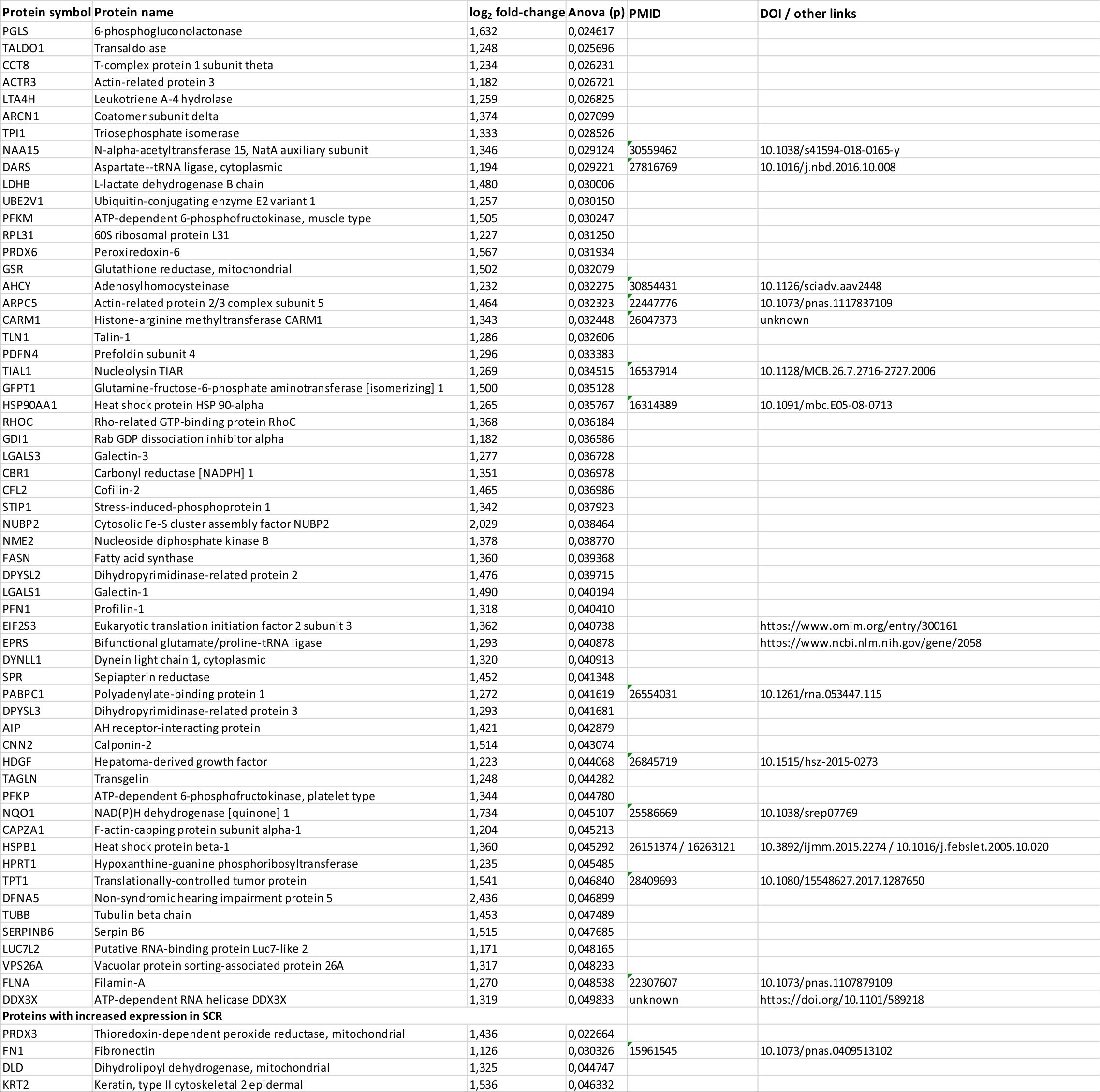
**

**Supplementary Figure 3 – continued**

**
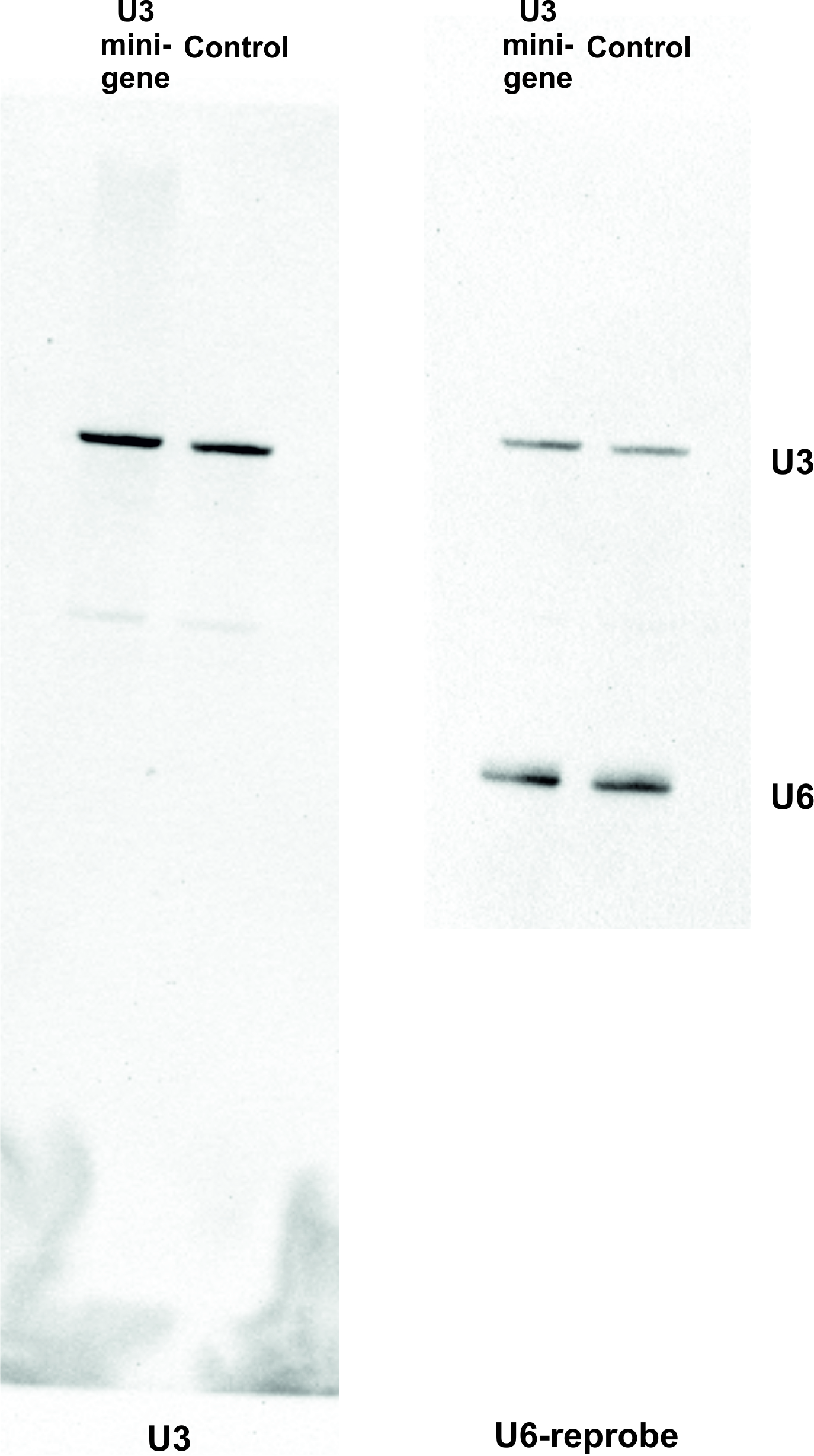
**

106 nt.

217 nt.

**Supplementary Figure 4:** Full lengths of northern blots depicted in Figure 3A. First the blot was probed with a U3-specific probe and imaged (left panel). This was followed by reprobing the same blot with a U6-specific probe. For quantification and compilation of Figure 3A, the left panel was used for U3 and the right panel was used for U6. The expected lengths of both RNAs are indicated.

**REFERENCES**

1 Balaskas, P. *et al.* MicroRNA Profiling in Cartilage Ageing. *International journal of genomics* **2017**, 2713725, doi:10.1155/2017/2713725 (2017).
